# Comparison between fast-interrupted steady-state (FISS) and rapid water-excitation pulses for fat signal suppression in free-running whole-heart MRI at 1.5 T

**DOI:** 10.1007/s10334-025-01273-z

**Published:** 2025-06-23

**Authors:** Yasaman Safarkhanlo, Jérôme Yerly, Mariana B. L. Falcão, Adèle L. C. Mackowiak, Davide Piccini, Matthias Stuber, Bernd Jung, Christoph Gräni, Jessica A. M. Bastiaansen

**Affiliations:** 1 Department of Cardiology, University Hospital Bern, Bern, Switzerland; 2 Translation Imaging Center (TIC), Swiss Institute for Translational and Entrepreneurial Medicine, Bern, Switzerland; 3 Graduate School for Cellular and Biomedical Sciences (GCB), University of Bern, Bern, Switzerland; 4 Department of Diagnostic and Interventional Radiology, Lausanne University Hospital (CHUV), University of Lausanne (UNIL), Lausanne, Switzerland; 5 Center for Biomedical Imaging, Lausanne, Switzerland; 6 Department of Diagnostic, Interventional and Pediatric Radiology (DIPR), Inselspital, Bern University Hospital, University of Bern, Bern, Switzerland; 7 Advanced Clinical Imaging Technology, Siemens Healthineers International AG, Lausanne, Switzerland

**Keywords:** Off-resonance, Fat suppression, 5D whole-heart free-running CMR, 1.5 T, LIBRE, FISS, bSSFP

## Abstract

**Background:**

Free-running whole-heart MRI using balanced steady-state free precession (bSSFP) sequences offer high SNR and myocardial tissue contrast. However, an inadequate fat signal suppression may introduce artifacts and is particularly challenging with non-Cartesian readouts. The aim of this study was to evaluate different fat-signal suppression methods for whole-heart free-running MRI at 1.5 T using numerical simulations, phantom, and cardiac MRI experiments without the use of contrast agents.

**Methods:**

Binomial off-resonant rectangular (BORR), lipid insensitive binomial off-resonant RF excitation (LIBRE), and lipid insensitive binomial off-resonant (LIBOR) pulses were implemented within a 3D radial bSSFP sequence. Their pulse parameters were optimized for fat signal suppression at 1.5 T using simulations and phantom experiments. Optimized protocols, along with a free-running fast interrupted steady-state (FISS) and non-fat suppressed bSSFP sequence, were used to acquire phantom and cardiac data in five volunteers. SAR values were recorded. The SNR and CNR_Water-Fat_ were measured in phantom data, while SNR and CNR_Blood-Myocardium_ were quantified in volunteers using reconstruction without motion correction. Motion-resolved reconstructions were used for qualitative assessments. Statistical differences were analyzed using one-way ANOVA.

**Results:**

LIBOR had the highest CNR_Water-Fat_ (276.8 ± 2.5) in phantoms, followed by LIBRE (268.1 ± 2.6), BORR (249.9 ± 2.2), and FISS (212.7 ± 2.7), though these differences were not statistically significant (*p* > 0.05). In volunteers, BORR had the highest SNR in the ventricular blood pool (17.0 ± 1.5), and LIBRE had the highest CNR_Blood-Fat_ (29.4 ± 9.3). FISS had the highest CNR_Blood-Myocardium_ (29.0 ± 8.9), but the differences were not significant (*p* > 0.05). Motion-resolved cardiac imaging showed comparable quality across all fat-suppressed sequences, with no significant streaking artifacts observed. Free-running bSSFP with LIBOR required the lowest SAR, up to a sixfold decrease compared with FISS.

**Conclusion:**

The tested sequences performed similarly in SNR and CNR but LIBOR offered the lowest SAR, making it a promising candidate for applications where RF energy deposition is a concern.

## Introduction

Cardiac MRI (CMR) typically necessitates skilled technicians for precise planning and requires cardiac triggering and patients performing multiple breath-holds [1]. Free-running whole-heart MRI greatly simplifies the acquisition process and eliminates the need for breath-holds [2]. Free-running MRI using a spiral phyllotaxis trajectory allows for the retrospective reconstruction of motion-resolved 5D whole-heart images for any motion state and spatial orientation [3]. However, the radial trajectories hinder the performance of conventional fat suppression techniques, often leaving residual fat signal.

To achieve a high signal-to-noise ratio (SNR) and blood-myocardium tissue contrast [4], balanced steady-state free precession (bSSFP) is typically preferred at 1.5 T [5, 6]. However, bSSFP imaging produces bright water and fat signals, which can impede the differentiation between pericardial fluid and fat, blurring the visualization of small anatomical structures, such as coronary arteries. To overcome these limitations, fat signal suppression techniques can be used, including chemical shift selective fat suppression [7], short inversion time inversion recovery [8], and specific magnetization preparation modules [9, 10]. The use of multi-echo approaches may lead to longer repetition times (TR) [11, 12] or introduce sensitivity to B0 inhomogeneity and partial-volume effects [13]. An inadequate fat signal suppression can lead to fat–water signal cancellation or India ink artifacts [14], obscuring vessel boundaries and other water–fat interfaces. It can also introduce streaking artifacts, particularly in cases where subcutaneous chest fat is not effectively suppressed. Due to the fast T1 recovery of fat signal and the k-space center-dominated MRI signal weighting in radial sampling, conventional fat suppression methods are challenging in advanced whole-heart MRI techniques [15, 16].

The first use of fat saturation in the free-running framework at 1.5 T demonstrated residual Indian ink artifacts and required prolonged scan times (up to 14 min) to comply with specific absorption rate (SAR) constraints, due to the SAR demand of both bSSFP and the fat saturation module [2]. Water excitation (WE) methods offer an alternative to fat saturation because they selectively excite water without relying on uniform fat suppression [17]. However, at lower magnetic field strengths, the resonance frequency difference between water and fat narrows, which increases the WE pulse durations and prolongs the acquisition time.

To address these limitations, advanced water excitation strategies [18–21] and fast interrupted steady-state (FISS) sequences [22] were introduced for rapid and robust fat suppression in whole-heart radial MRI. In FISS, the bSSFP steady state is interrupted to create a wide signal stop-band centered around the main fat resonance [22–24], which can be modified by changing the repetition time TR. For example, the optimal TR for fat signal suppression lies between 2.0 ms and 3.0 ms at 1.5 T, and between 3.0 and 3.5 ms at 3 T, both with eight readouts per FISS module [22]. This TR range may impact the achievable spatial resolution or the efficiency of fat signal suppression at 1.5 T. For coronary angiography a high spatial resolution is needed, which resulted in a minimum TR of 3.2 ms for free-running FISS at 1.5 T [22].

To address the extended duration of conventional water excitation pulses, off-resonant water excitation pulses were proposed, such as the binomial off-resonant rectangular (BORR) [25, 26] and the lipid insensitive binomial off-resonant RF excitation (LIBRE) [18, 19, 27]. These RF pulses have a flexible RF pulse duration that can be shortened at the expense of an increased RF power [20, 27], resulting in a higher RF energy deposition. Although shorter compared with conventional water-excitation, they significantly improved fat signal suppression and have been applied in various imaging applications [18, 19, 28–34]. However, incorporating such pulses in a SAR-intensive bSSFP sequence can present practical challenges. For instance, in a whole-heart free-running bSSFP acquisition at 1.5 T using LIBRE pulses, the optimal contrast between the blood pool and myocardium was affected by the SAR constraints [19], highlighting the difficulty of balancing effective fat suppression and image quality. Recently, an RF-power-optimized off-resonant water excitation pulse, called lipid insensitive binomial off-resonant (LIBOR) pulse, was proposed for whole-heart MRI at 3 T [20]. LIBOR reduced SAR in comparison with LIBRE and BORR while maintaining robust fat suppression [20]. This makes this pulse particularly suitable for SAR-intensive acquisitions, such as free-running bSSFP at 1.5 T. Similar to LIBRE and BORR, the LIBOR pulse offers a flexible RF pulse duration, which can be shortened by changing the RF pulse properties. The potential of LIBOR pulses within a free-running bSSFP sequence for whole-heart MRI at 1.5 T is promising, because it simultaneously addresses both SAR minimization and fat signal suppression.

Fat suppression remains a challenge for 3D radial bSSFP sequences, particularly in whole-heart imaging at clinical 1.5 T scanners. Despite different developments for fat signal suppression, they have not been systematically compared in free-running whole-heart MRI sequences. Therefore, the aim of the study was to implement the LIBOR water-excitation pulse in a free-running bSSFP sequence at 1.5 T and to perform a comprehensive comparison with BORR and LIBRE water-excitation pulses, as well as FISS. The fat suppression capabilities, SAR deposits, and other relevant metrics were quantified for each method in phantom and healthy volunteer experiments. By doing so, this study tried to identify the method that achieves the highest SNR while minimizing SAR, ensuring optimal image quality for clinical applications.

## Methods

### Pulse sequence design and implementation

**Fig. 1:**
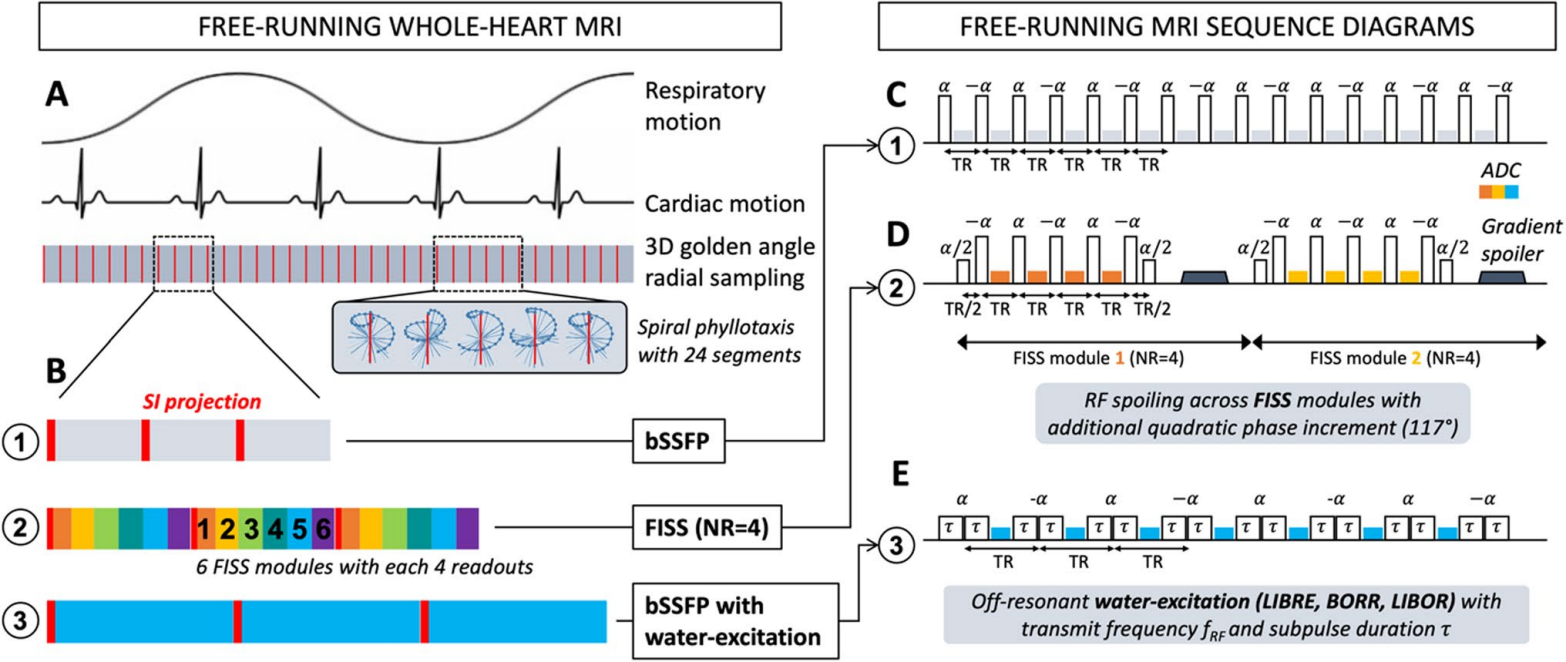
Schematic overview of the free-running MRI sequences. Data acquisition is performed continuously, irrespective of cardiac and respiratory motion (**A**). Different acquisitions for fat signal suppression were performed (**B**); a free-running bSSFP sequence with standard rectangular excitation pulses (**C**), a FISS sequence with standard rectangular excitation pulses (**D**), a bSSFP sequence with BORR, LIBRE, and LIBOR water excitation pulses (**E**). Numerical simulations were performed to tune the RF pulse parameters, which were experimentally verified

Data are continuously acquired in free-running MRI, irrespective of cardiac and respiratory motion (Fig. 1A). Different methods for fat signal suppression were evaluated (Fig. 1B), a standard bSSFP acquisition (Fig. 1C), a FISS acquisition (Fig. 1D), and three different types of off-resonant water excitation RF pulses integrated in a bSSFP acquisition (Fig. 1E). Both the free-running bSSFP and FISS sequences use nonselective rectangular RF pulses with a duration of 0.3 ms.

The FISS sequence is an interrupted bSSFP sequence, dividing it into FISS modules, as described in [35]. A FISS module starts with a ramp-down pulse (*α*/2) and ends with a magnetization restoration pulse (*α*/2). Four readouts were acquired per FISS module (NR = 4), a 117° quadratic RF spoiling was applied across FISS modules, and gradient spoilers were inserted after each FISS module. Note that all described implementations are embedded within the same MRI sequence.

As described previously, the LIBOR [20], BORR [20, 25], and LIBRE [27] RF pulses follow a binomial 1-1 pattern, consisting of two subpulses with variable subpulse duration τ. For all three pulses, shortening the RF pulse duration requires an increase in the off-resonance frequency. As a result, the RF power needs to increase to excite the water magnetization to the desired rotation angle. The main differences between these three water excitation pulses are the phase offset of the second subpulse Δφ_2_ and the required RF power, which changes their SAR signature.

More specifically, the BORR pulse is defined by a fixed 180° phase modulation on the second subpulse [25, 26]. The LIBRE pulse has a phase offset that depends on the subpulse duration and the RF pulse frequency Δ*φ*_2_ = 2πf_RF_τ [27]. To reduce RF power, the LIBOR pulse is defined by an RF pulse frequency that is half that of the LIBRE pulse, after which the phase offset is adjusted to achieve fat signal suppression [20]. So far, only the LIBRE pulse has been used at 1.5 T [19], for which the subpulse duration was set to 1.3 ms, leading to a total RF duration of 2.7 ms. Therefore, in the current study, the duration of all pulses was standardized to this value for a fair comparison.

To determine the LIBOR phase offset, numerical simulations of the Bloch equations were performed, which was subsequently verified in phantom experiments. The transverse magnetization *M*_*xy*_ as a function of the phase offset and off-resonance after a single LIBOR RF excitation was simulated using an RF excitation angle of 50° and an off-resonance frequency of 270 Hz. This frequency is half that of the 540 Hz off-resonance frequency of the LIBRE pulse at 1.5 T, which was found based on an iterative simulation. The off-resonance was varied between − 800 Hz and 800 Hz in steps of 10 Hz, and the phase offset was varied between 0° and 360° in steps of 10°. The phase offset corresponding to the widest signal suppression band around fat (− 220 Hz), defined as 10% of the maximum observed transverse magnetization (*M*_*xy*_), was selected for subsequent measurements. All simulations were performed in MATLAB 2021 (The MathWorks, Natick, MA, USA). The code for this simulation can be found on GitHub.

Another set of simulations was performed to calculate the RF power adjustment for all RF pulses. This was done by simulating the RF pulse response, meaning the transverse magnetization *M*_*xy*_ as a function of off-resonance following a single RF pulse excitation using either a LIBOR, BORR, LIBRE, or a single nonselective rectangular pulse. This RF response was then used to calculate the RF power adjustment to ensure a 50° rotation of the water magnetization, referred to as the nominal RF excitation angle. The off-resonance was varied between − 800 Hz and 800 Hz in steps of 10 Hz; the off-resonance frequency of LIBRE was set to 540 Hz, that of LIBOR to 270 Hz, BORR to 500 Hz, and the standard rectangular pulses to 0 Hz. The BORR transmit frequency of 500 Hz was chosen based on its optimal signal suppression at – 220 Hz. The results of these simulations informed on RF power increases for subsequent MRI experiments, which were achieved by increasing the RF excitation angle in the user interface of the MRI sequence. The RF waveforms corresponding to each of the RF pulses were plotted for comparison.

### Numerical simulations of free-running FISS and bSSFP sequences with and without off-resonant water excitation pulses

The steady-state transverse magnetization *M*_*xy*_ was simulated as a function of RF excitation angle and off-resonance for a bSSFP and a FISS sequence with nonselective RF excitation pulses, as well as a bSSFP sequence with LIBOR, LIBRE, and BORR RF excitation pulses. The RF properties found in the previous section were used (Table 1). RF excitation angles were varied around their previously determined optimal RF excitation angle by − 20° to + 20° in steps of 5°. The off-resonance was varied between − 800 Hz and 800 Hz in steps of 10 Hz. The repetition time (TR) was set to the minimum possible at the MRI scanner and was 2.47 ms for FISS and bSSFP and 4.9 ms for the bSSFP sequence with integrated LIBRE, BORR, and LIBOR pulses. A *T*_1_ of 1390 ms and *T*_2_ of 300 ms was used to reflect the relaxation times of blood at 1.5 T. To ensure a magnetization steady state, 1000 RF excitations were simulated. In the case of FISS, perfect RF and gradient spoiling were simulated by nulling the *M*_*xy*_ after each FISS module. The result of these simulations provides a comparison of water excitation efficiency, i.e., the required increase in power deposit relative to on-resonant pulses, fat signal suppression, and corresponding suppression bandwidths.

**Table 1:** MRI acquisition parameters and RF pulse properties

	bSSFP	FISS	BORR	LIBRE	LIBOR
Total RF duration (ms)	0.3	0.3	2.7	2.7	2.7
Subpulse duration τ (ms)	/	/	1.3	1.3	1.3
TR/TE (ms)	2.47/1.24	2.47/1.24	4.90/2.46	4.90/2.46	4.90/2.46
FOV (mm^3^)	220^3^	220^3^	220^3^	220^3^	220^3^
RF frequency offset (Hz)	0	0	500	540	270
RF excitation angle at scanner user interface (°)	50	50	141	124	78
Nominal RF excitation angle α (°)	50	50	50	50	50
RF phase offset Δφ_2_ (°)	/	/	180	253	290
Receiver bandwidth (Hz/Px)	992	992	992	992	992
Acquisition time	1 min 51 s	2 min 55 s	3 min 41 s	3 min 41 s	3 min 41 s

Five different acquisitions were conducted in this study: A free-running bSSFP sequence with standard rectangular excitation pulses, a FISS sequence with standard rectangular excitation pulses, a bSSFP sequence with BORR, LIBRE, and LIBOR water excitation pulses. Each scan was performed with tuned pulse parameters, including RF frequency offset, excitation angle, and phase offset, to achieve the best fat suppression performance. The nominal RF excitation angle refers to the rotation angle of the water magnetization, while the RF excitation angle represents the value displayed and adjusted by the user in the scanner user interface. The latter aspects reflect the different RF power requirements and SAR intensities (Fig. 7)

### Validation of fat signal suppression in a fat phantom

To validate fat signal suppression, MRI experiments were performed using a 3D radial free-running bSSFP MRI sequence with different off-resonant water excitation methods on a 1.5 T clinical MRI scanner (MAGNETOM Sola, Siemens Healthcare, Erlangen, Germany). Each experimental setup was repeated three times over three separate days. The fat phantom contained vials with different fat percentages, ranging from 0 to 100% of water and peanut oil mixtures, as described in [36].

The study included two independent sets of experiments. First, the RF excitation frequency offset, RF excitation angle, and LIBOR phase offset were varied systematically to compute water and fat contrast in the resulting images. The initial parameters were guided by the results of the numerical simulations. In the second experiment, the effectiveness of fat signal suppression for every pulse was compared using the RF parameters from the first phantom experiment. The FISS sequence was also included in this comparison, with its parameters taken from our prior work [22]. Note that the exact same FISS sequence was used in the current study, which allows switching to bSSFP and switching between RF excitation pulses from the user interface.

To test the effect of RF excitation frequency offset on the measured signal, the RF excitation angle was kept constant. For BORR, with a fixed RF excitation angle of 141°, the frequency was varied from 400 to 600 Hz in steps of 20 Hz. For LIBRE, with an RF excitation angle of 124°, the same frequency range was used. For LIBOR, with an RF excitation angle of 78° and a phase offset of 285°, the frequency was varied from 250 to 340 Hz in 10 Hz increments.

To test the effect of RF excitation angle on the measured signal, the RF excitation frequency was held constant. For BORR, the RF excitation angles were varied in 10° increments from 90° to 180°, while keeping the frequency fixed to 600 Hz, using a frequency offset of 600 Hz. For LIBRE, the RF excitation angles were varied from 50° to 180° in steps of 10°, using a frequency of 500 Hz. For LIBOR, with a frequency offset of 270 Hz and a phase offset of 285°, RF excitation angles ranged from 50° to 100° in steps of 5°.

Finally, to determine the effect of the LIBOR phase offset on the water-fat contrast, a phase offset sweep from 250° to 340°, with steps of 10°, was performed. The LIBOR pulse using frequency was 270 Hz with an RF excitation angle of 78°.

Further acquisition parameters were constant and included: a field of view (FOV) of 220 × 220 × 220 mm^3^, matrix size of 112 × 112 × 112, a  (2.0 mm)^3^ isotropic resolution, a 992 Hz/pixel bandwidth (BW). The radial trajectory used 24 lines per interleaf with 246 interleaves for a total of 5904 radial readouts, efficiently sampling the entire 3D k-space to ensure comprehensive volumetric data acquisition. The echo time (TE) and repetition time (TR) varied per experiments (Table 1).

The phantom results were used to decide on the RF parameters for the whole-heart MRI experiments (Table 1). This final protocol was tested in a phantom with an increased number of radial readouts (24 × 1877). A FISS acquisition was added for comparison.

### Contrast-free 5D whole-heart free-running MRI in volunteers

Five healthy volunteers (*n* = 5; 4 females; 27.4 ± 7.5 years old; height 168.8 ± 7.8 cm; weight 64.4 ± 10.9 kg) participated in this study, who provided written and informed consent approved by our local ethical review board.

The volunteer’s protocol was based on the ECG-triggered non-interrupted 3D radial free-running bSSFP sequence, as used in the comparison setup for the phantom experiment (Table 1). The data were acquired on a 1.5 T clinical MRI scanner (MAGNETOM Sola, Siemens Healthcare, Erlangen, Germany) with four fat suppression methods (FISS, BORR, LIBRE, and LIBOR), along with a bSSFP sequence without any fat signal suppression. This comparison followed a similar setup described in a previous study by Masala et al. [19], where a 32-channel spine coil and an 18-channel chest coil were used during the free-breathing acquisition. The radial readouts were arranged in interleaves based on a spiral phyllotaxis pattern, with successive interleaves rotated about the z-axis by the golden angle, and the first readout in each interleave oriented in the superior-inferior (SI) direction for subsequent physiological motion extraction and binning into cardiac and respiratory phases.

Because of differences in RF pulse duration, the TR was 2.47 ms for bSSFP and FISS, and 4.9 ms for LIBRE, LIBOR, and BORR. The total number of acquired radial lines was constant using a spiral phyllotaxis trajectory consisting of 1877 spirals, with each 24 k-space segments. The resulting acquisition time for each sequence was constant for all volunteers, independent of their respiratory pattern and heart rates, with 3 min and 41 s for BORR, LIBRE, and LIBOR, 2 min and 55 s for FISS, and 1 min and 51 s for the bSSFP sequence without fat suppression.

### 5D whole-heart motion resolved volunteer image reconstruction

The volunteer data were reconstructed using an in-house MATLAB code, following the free-running framework described by Di Sopra et al. [37]. Two separate reconstructions were performed: first, a reconstruction without motion correction to maintain the noise properties for quantitative analysis, specifically for SNR and contrast-to-noise ratio (CNR) comparisons. This reconstruction used radially sampled k-space data with coil sensitivity combination, implemented using the gpuNUFFT library. The coil sensitivity maps were estimated using ESPIRiT from auto-calibrated signal (ACS) data embedded within the free-running acquisition, following the reconstruction pipeline described by Di Sopra et al. [37]. The offline pipeline includes density compensation, NUFFT-based reconstruction without motion correction, and iterative SENSE reconstruction. Second, a compressed sensing reconstruction was carried out to generate 5D cardiac and respiratory motion-resolved images. This was achieved using the alternating direction method of multipliers (ADMM) algorithm [37, 38].

To enhance the quality of image reconstruction and improve local low-rank (LLR) representations, several regularization techniques are possible, including total variation in the cardiac (TVt), respiratory (TVr), and spatial (TVs) dimensions, as well as local low-rank constraints on cardiac (LRtWeight) and respiratory dimensions (LRrWeight). The regularization weights were optimized through a grid search to achieve the best compromise between preserving image detail and minimizing noise. The selected weights were set as follows: TVsWeight = 0, TVtWeight = 0.01, TVrWeight = 0.01, LRtWeight = 0.05, and LRrWeight = 0.05. This combination effectively suppressed artifacts and noise while maintaining both the spatial and temporal resolution of the reconstructed images, which were then used for qualitative assessments of cardiac and respiratory motion.

The motion-resolved reconstruction included four respiratory phases and 25 cardiac phases, allowing detailed visualization of both cardiac and respiratory motion throughout the imaging volume.

The reconstruction time for each dataset was approximately 3 h, executed using MATLAB R2022b on an Ubuntu 22.04.3 workstation equipped with two 32-core CPUs (AMD Ryzen Threadripper 2990WX, Santa Clara, CA), 128 GB of RAM, and an NVIDIA GeForce RTX 2080 Ti (Nvidia, Santa Clara, CA).

### Data analysis

Phantom MRI data were reconstructed at the scanner using the vendor-provided pipeline, including predefined coil combinations and gridding optimized for radial trajectories. These phantom images were processed as a single phase without binning or temporal regularization. Because the inline reconstruction does not support motion-resolved or compressed sensing methods, the offline reconstructions were performed in MATLAB as described by Di Sopra et al. [37]. The SNR was calculated in selected regions of interest (ROIs) on the same fat fraction vial in each acquisition, ensuring consistent relative positioning across all images. For SNR measurements, the noise standard deviation (SD) was measured in background regions carefully selected to be free from structured artifacts, residual aliasing, or streaking. Multiple noise ROIs were assessed across different acquisitions to verify consistency in background noise distribution. While coil sensitivity estimation in signal-free regions has inherent limitations, this approach provides a reasonable approximation of system noise when regions are appropriately chosen. For signal measurements, ROIs were placed well within homogeneous regions of the phantom and tissues, avoiding boundaries where potential Gibbs ringing or partial volume effects could influence measurements. SNR was then calculated as the ratio of the mean signal within the ROI to the SD of the background noise. All measurements were conducted using ImageJ software (National Institutes of Health, Wisconsin University, Bethesda, MD), with uniform brightness and contrast settings applied to maintain consistency across images. For CNR calculations, the difference in SNR between two distinct ROIs was computed.

In the volunteer studies, SNR and CNR calculations were based on 3D reconstruction without motion correction to retain original noise properties. SNR was measured using ROIs drawn in the septal myocardium, left ventricular blood pool, chest fat, right lung, and background, in the axial view, being visually chosen for ROI placement. The CNRs were calculated by subtracting the blood pool SNR from the chest fat SNR (CNR_Blood-Fat_) and the myocardium SNR (CNR_Blood-Myocardium_). For the 5D motion-resolved images, only qualitative assessments were performed, as the compressed sensing (CS) reconstruction altered the noise characteristics, rendering standard SNR and CNR measurements unsuitable. Since these analyses focused on objective, quantitative measurements rather than subjective evaluations, no additional blinding to pulse sequences was deemed necessary.

For both phantom and volunteer studies, the specific absorption rate (SAR) values, representing RF energy absorption levels, were retrieved from the DICOM files. These values were compared between different acquisitions to evaluate the relative RF energy absorption levels.

### Statistical analysis

All results are reported as means ± standard deviations. A one-way analysis of variance (ANOVA) was conducted to evaluate differences in SNR and CNR between the pulse sequences, with statistical significance set at *p* < 0.05. If significant differences were detected, Tukey’s Honestly Significant Difference (HSD) test was applied as a post-hoc analysis to identify pairwise differences while controlling for Type I errors.

SAR values were measured for each sequence and reviewed separately to assess RF energy absorption. The optimal pulse sequence was selected based on a combined assessment of high SNR and low SAR. The sequence demonstrating the best balance between these two factors was identified for further applications.

## Results

### RF pulse design and numerical simulations

**Fig. 2:**
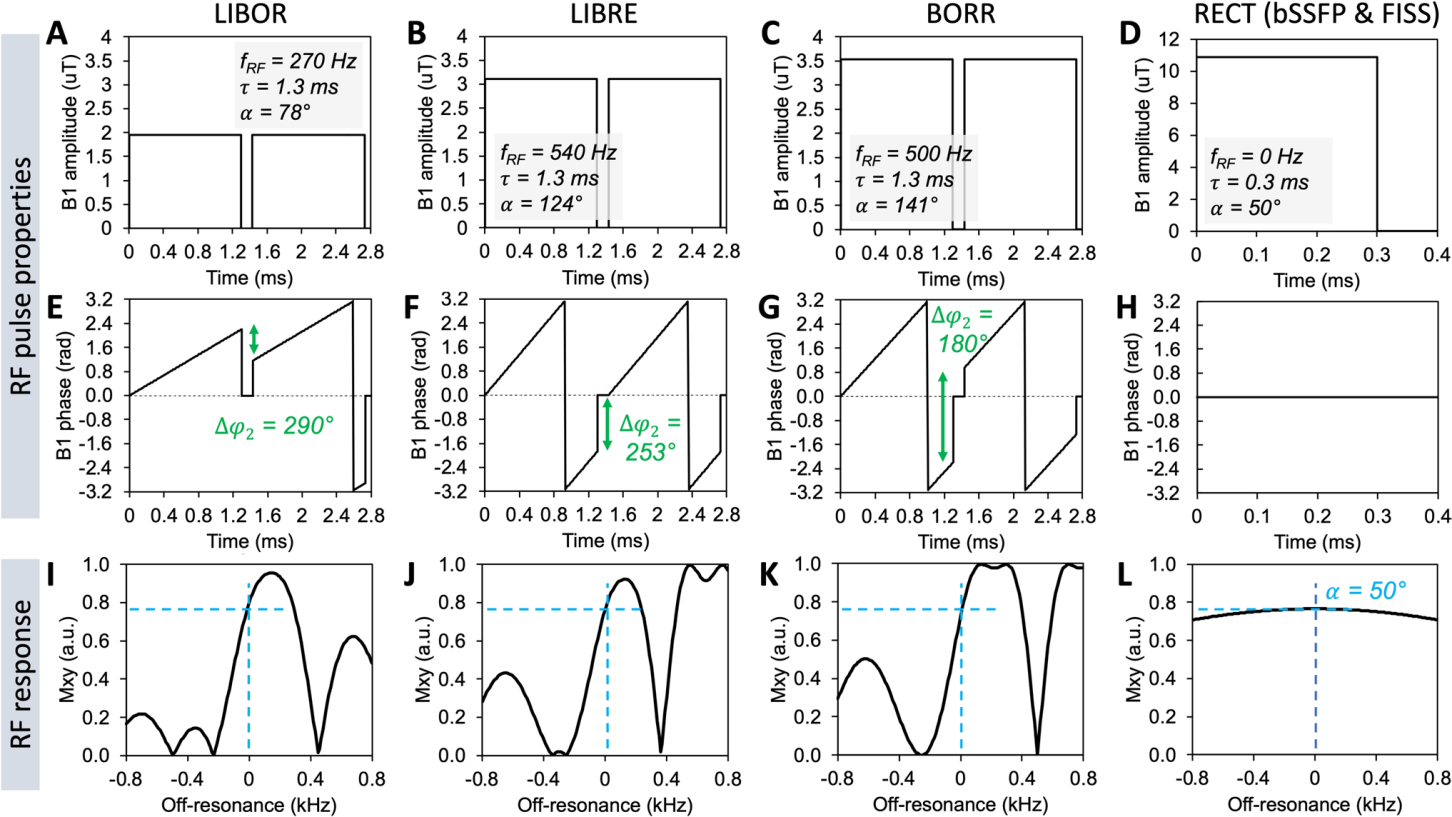
RF pulse shapes and RF pulse response of the LIBOR, LIBRE, and BORR water excitation pulses and a standard rectangular RF pulse. The RF pulse waveforms illustrate the B1 amplitude (**A**–**D**) and B1 phases (**E**–**H**) of the different water excitation pulses to achieve the same excitation of water at 0 Hz (**I**–**L**). The RF excitation frequency corresponds to the slope of the B1 phase, while the phase offset Δφ_2_ corresponds to the difference in B1 phase between the end of the first subpulse and the start of the second subpulse. RF pulse properties were either previously defined or set through numerical simulations in the current study. The RF response, the transverse magnetization as a function of off-resonance, demonstrates that fat signal suppression around -220 Hz is achieved for all water-excitation pulses, requiring different RF powers to achieve a nominal RF excitation angle of 50°﻿ for the water magnetization (blue dashed lines)

The RF pulses show distinct properties (Fig. 2). Simulations of the LIBOR pulse indicated that for optimal fat signal suppression, the RF phase offset of the second subpulse was between 270° and 310°. Using a phase offset of 290° and an excitation frequency offset of 270 Hz, the RF pulse power required an increase of 1.56 (RF excitation angle of 78°) to achieve a 50° rotation of the water magnetization with respect to an on-resonant excitation pulse (Fig. 2AD). The power increase was 2.48 and 2.82 for LIBRE and BORR, respectively (Fig. 2B, C). The RF response of all tested pulses, including their optimal parameters, shows fat signal suppression around − 220 Hz and the same level of water excitation around 0 Hz (Fig. 2I–L).

**Fig. 3:**
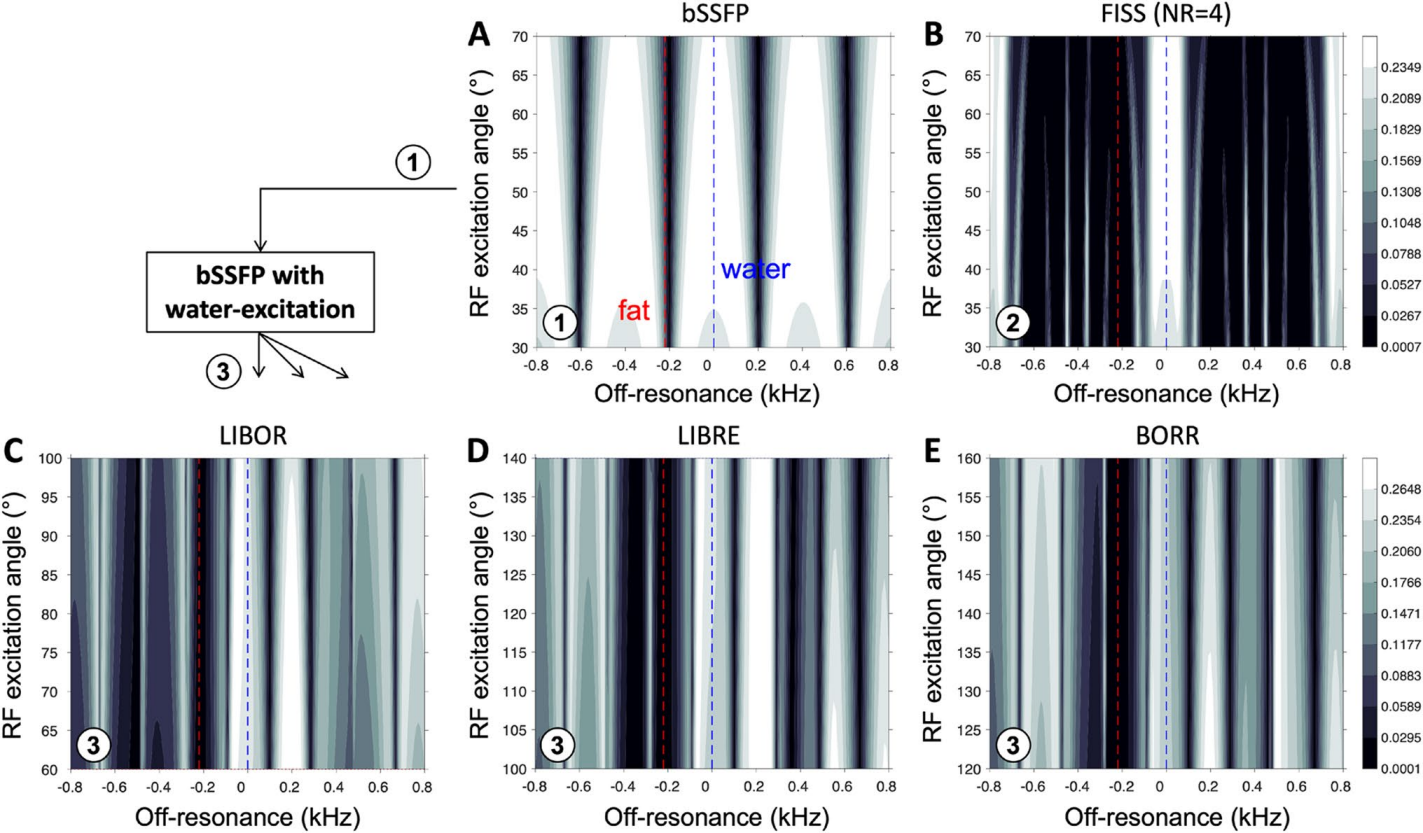
Steady-state magnetization of free-running FISS and bSSFP with and without off-resonant water excitation pulses. Simulations of a bSSFP sequence without (**A**) and with (CDE) the three off-resonant pulses, as well as a FISS sequence (**B**), illustrate the transverse magnetization *M*_xy_ as a function of RF excitation angle and off-resonance. Simulations demonstrate the water excitation around 0 Hz (blue dashed line), and fat suppression around -220 Hz as expected (red dashed line). The TR of FISS and bSSFP was different (TR = 2.47ms) compared with those of bSSFP with water excitation (TR = 4.9ms), resulting in slightly lower transverse magnetization values. Furthermore, simulations indicate a certain robustness to B1 inhomogeneity as the transverse magnetization does not vary significantly across the simulated RF excitation angles. Note that a nominal water excitation of 50° is achieved at the center of each RF excitation range

Simulations of the steady-state magnetization of a bSSFP and FISS sequence using nonselective RF excitation pulses (Fig. 3A, B) show a slightly lower transverse magnetization compared with a bSSFP sequence using water excitation (Fig. 3C–E), due to a decrease in TR (Table 1). Furthermore, simulations indicate a certain robustness to B1 inhomogeneity as the transverse magnetization does not vary significantly across the simulated RF excitation angles. Signal suppression of the main fat resonance is observed around − 220 Hz, even for the bSSFP sequence using rectangular pulses. However, the fat signal suppression bandwidth increases to similar levels (~ 100–150 Hz) when using FISS or the water excitation pulses. Although the suppression bandwidths appear similar, the magnetization response as function of off-resonance are not comparable. Therefore, further validation in an experimental setting is required, because the fat spectrum contains multiple resonance peaks, the magnetic field is not necessarily homogeneous, and motion of the heart may disrupt the magnetization steady state.

### Fat signal suppression performance in phantom experiments

**Fig. 4:**
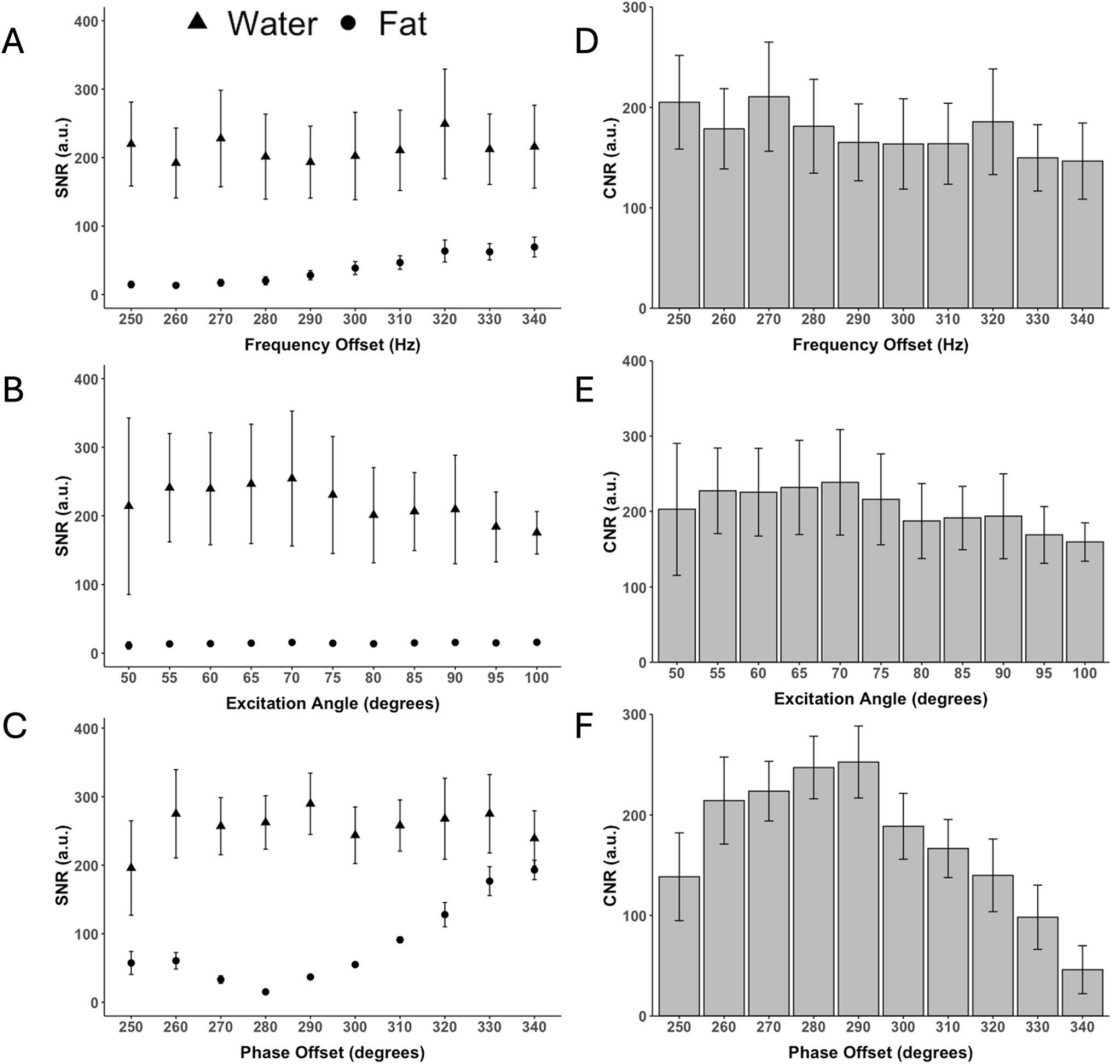
Fat and water signal-to-noise (SNR) and contrast-to-noise (CNR) ratios for LIBOR pulse across varying RF parameters optimization in a fat phantom. The SNR for (**A**) RF excitation frequency offsets, (**B**) RF excitation angles, and (**C**) phase offsets, with corresponding water-fat CNR for (**D**–**F**). All tested RF pulse parameters, besides the RF phase offset, suppress fat signal to a similar degree, indicating robustness to small variations in RF pulse settings. Optimal fat suppression is achieved with a frequency offset of 270 Hz, RF excitation angle of 70–80°, and phase offset of 290°

**Fig. 5:**
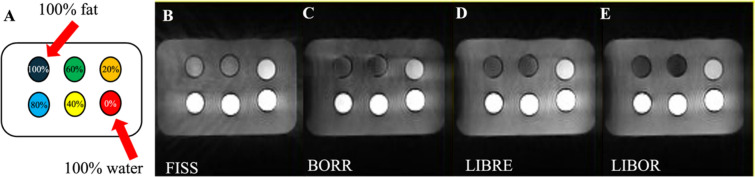
MRI experiment with various methods for fat signal suppression in a fat phantom. (**A**) Phantom configuration featuring a 100% fat vial in the top left and 100% water in the bottom right corner, highlighted by the red arrow. All approaches facilitate fat suppression to a certain degree, and comparable image contrast can be achieved for the different excitation schemes by modifying the RF excitation angle. (**B**) FISS pulse with an RF excitation angle of 50°. (**C**) BORR pulse with a frequency of 500 Hz and an RF excitation angle of 141°. (**D**) LIBRE pulse with a frequency of 540 Hz and an RF excitation angle of 124°. (**E**) LIBOR pulse with a frequency offset of 270 Hz, an RF excitation angle of 78°, and a phase offset of 290°. Contrast window and level settings are the same in all images

Using LIBOR, maximum CNR between water and fat was achieved using an RF excitation frequency offset of 270 Hz, a 70° RF excitation angle, and a 290° phase offset (Fig. 4). For BORR, the highest CNR was obtained with a 500 Hz RF excitation frequency offset and an RF excitation angle of 140°, while for the LIBRE pulse, the optimal settings were a frequency of 540 Hz and a 120° RF excitation angle. The performance of each pulse acquisition in the phantom experiments was visually comparable, with similar brightness levels observed across all acquisitions in vials containing different fat percentages for each pulse sequence (Fig. 5).

**Fig. 6:**
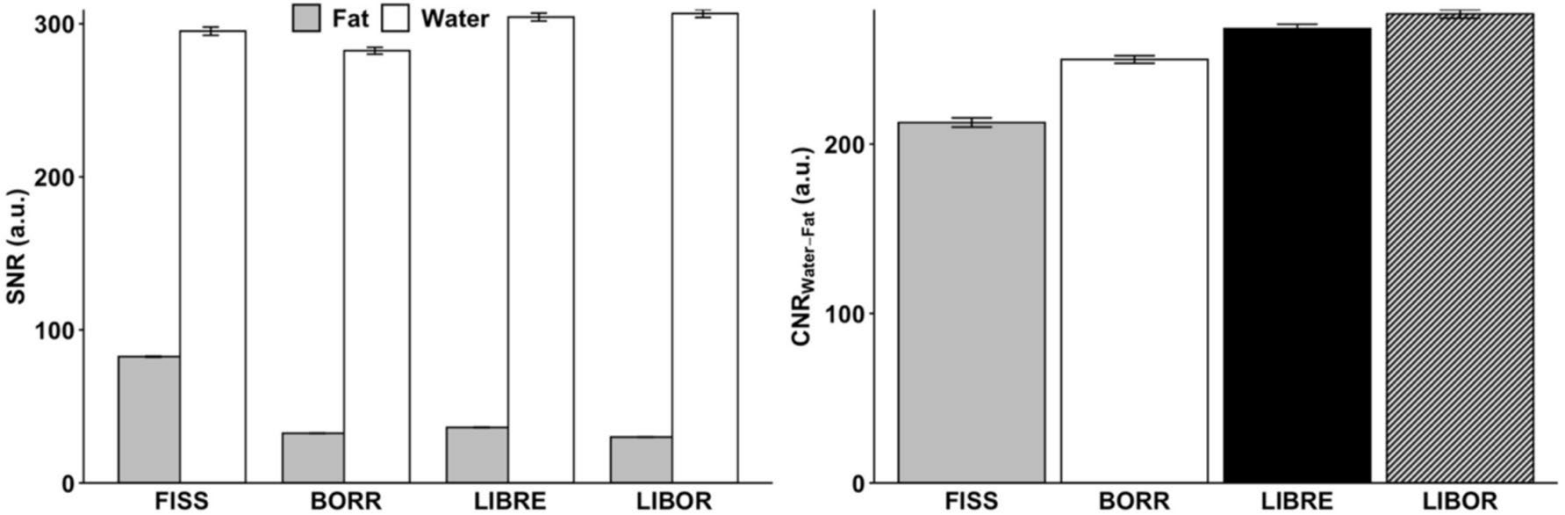
Signal-to-noise ratio (SNR) and contrast-to-noise ratio (CNR) comparison for different fat suppression methods in phantom experiments. (**A**) Comparison of SNR and (**B**) CNR between water and fat for four distinct fat signal suppression methods, FISS, BORR, LIBRE, and LIBOR. In the SNR figure, grey denotes fat, and white represents water’s SNR

Comparing CNR between water and fat for different fat suppression pulses, LIBOR (276.8 ± 2.5) exhibited the highest value, while FISS (212.7 ± 2.7) had the lowest. The CNR values for BORR and LIBRE were 249.9 ± 2.2 and 268.1 ± 2.6, respectively (Fig. 6).

**Fig. 7:**
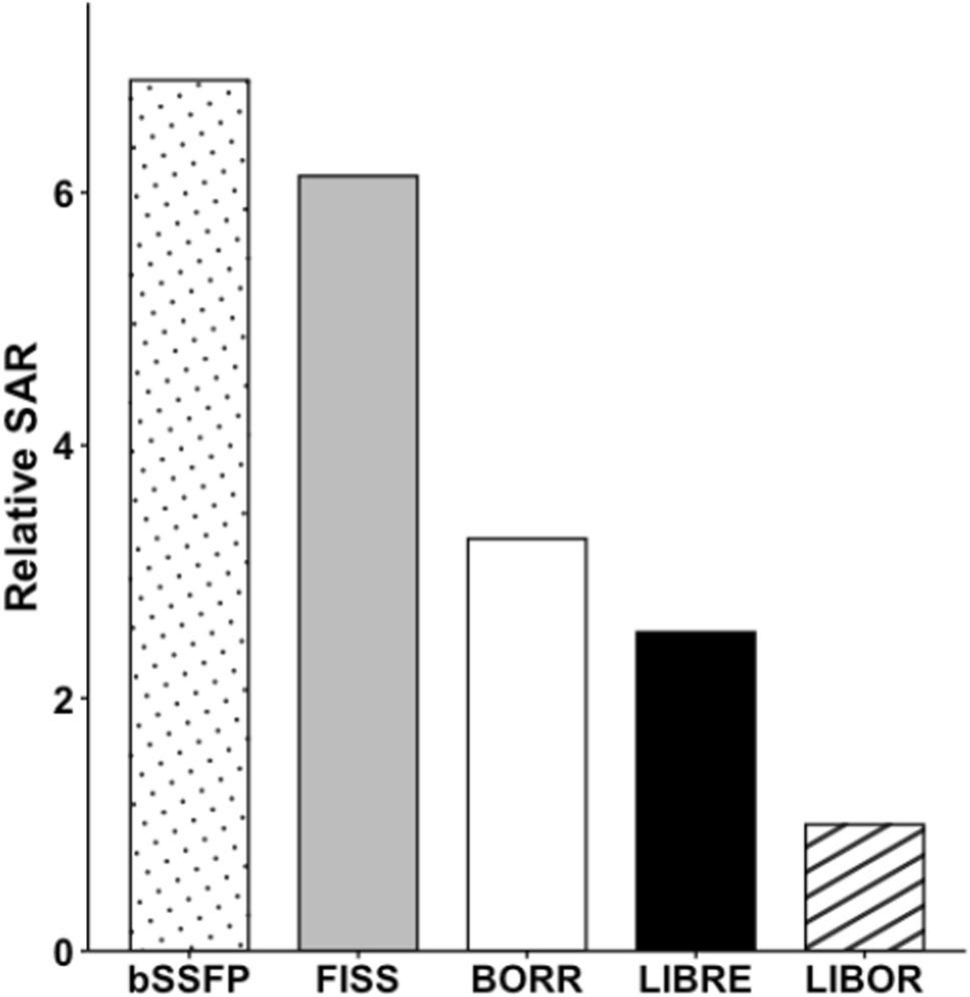
Specific absorption rate (SAR) for free-running whole-heart MRI sequences using different fat signal suppression methods. Relative SAR values of the different free-running whole-heart MRI sequences tested in the study. A regular free-running bSSFP sequence with standard rectangular pulses was compared with a free-running FISS implementation, as well as a free-running bSSFP sequence with three different water-excitation RF pulses, BORR, LIBRE, and LIBOR. The LIBOR water-excitation pulse required the lowest SAR

In terms of RF energy deposition, the SAR values showed that FISS deposited 6.1 times, BORR 3.3 times, and LIBRE 2.5 times more energy than LIBOR (Fig. 7).

### Fat suppression pulse performance in volunteer experiments using a contrast-free 5D whole-heart free-running bSSFP sequence

**Fig. 8:**
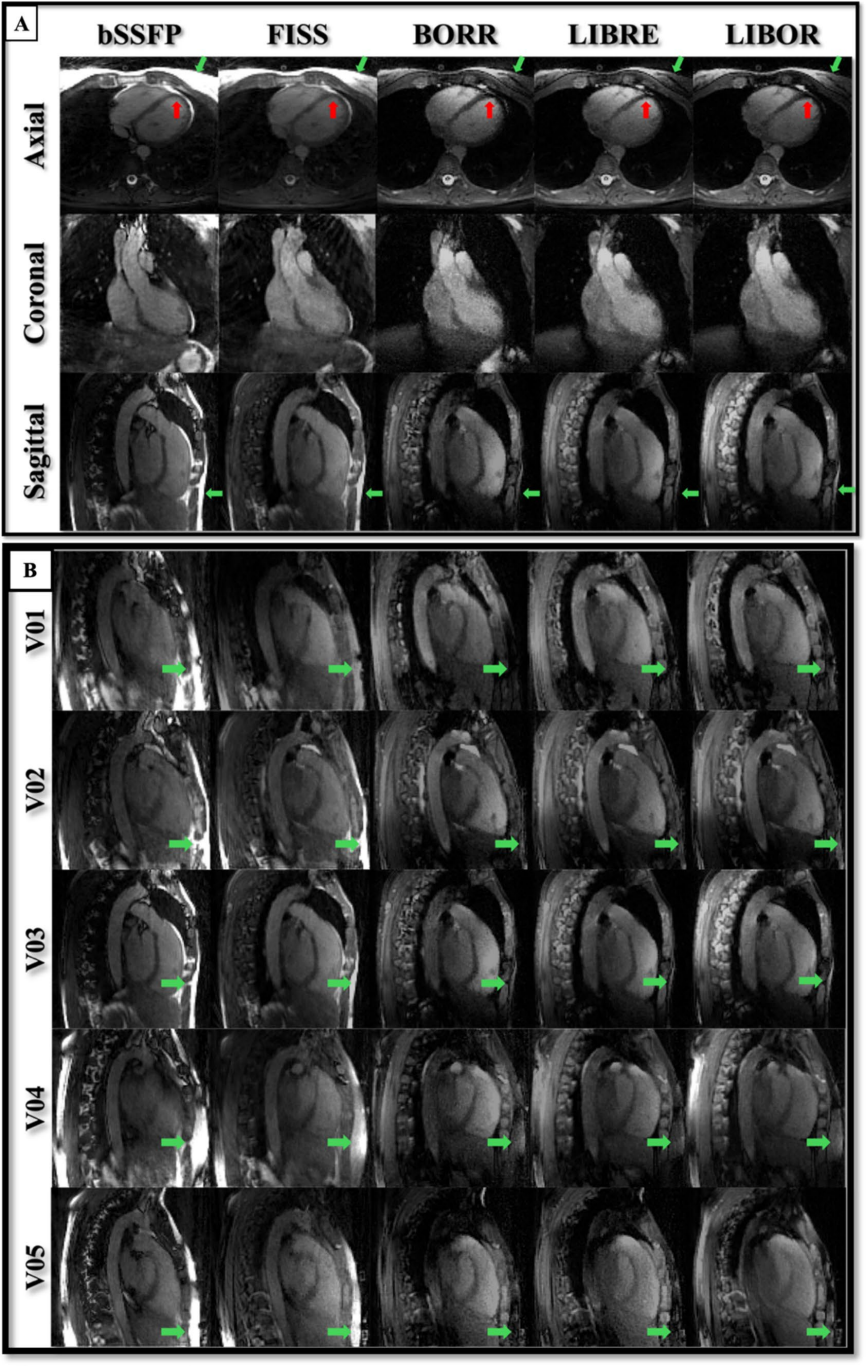
Cardiac and respiratory motion‐resolved 5D whole‐heart MRI reconstructed for volunteer data acquired with different fat suppression methods. (**A**) Representative images from one volunteer showing conventional non-fat-suppressed bSSFP, FISS, BORR-bSSFP, LIBRE-bSSFP, and LIBOR-bSSFP acquisitions. The images display the heart in the end-diastolic phase of the cardiac cycle and the end-expiration phase of the respiratory cycle. Red arrows show the myocardium wall, and green arrows represent subcutaneous fat. (**B**) Comparison of LIBOR-bSSFP acquisitions across all five volunteers (V01–V05), demonstrating consistent fat suppression performance across different subjects. Note that the perceived CNR is slightly reduced compared to previous studies due to the nominal RF excitation angle being limited to 50° in all volunteer experiments. The contrast window and level settings are consistent within each panel

In the volunteer study, the performance of each acquisition was visually comparable, with similar brightness levels observed in the myocardium, left ventricular blood pool, chest fat, right lung, and background in the 5D motion-resolved reconstructed images (Fig. 8). No significant streaking artifacts were observed in the volunteer scans across the sequences with fat suppression, FISS, BORR, LIBRE, and LIBOR.

**Fig. 9:**
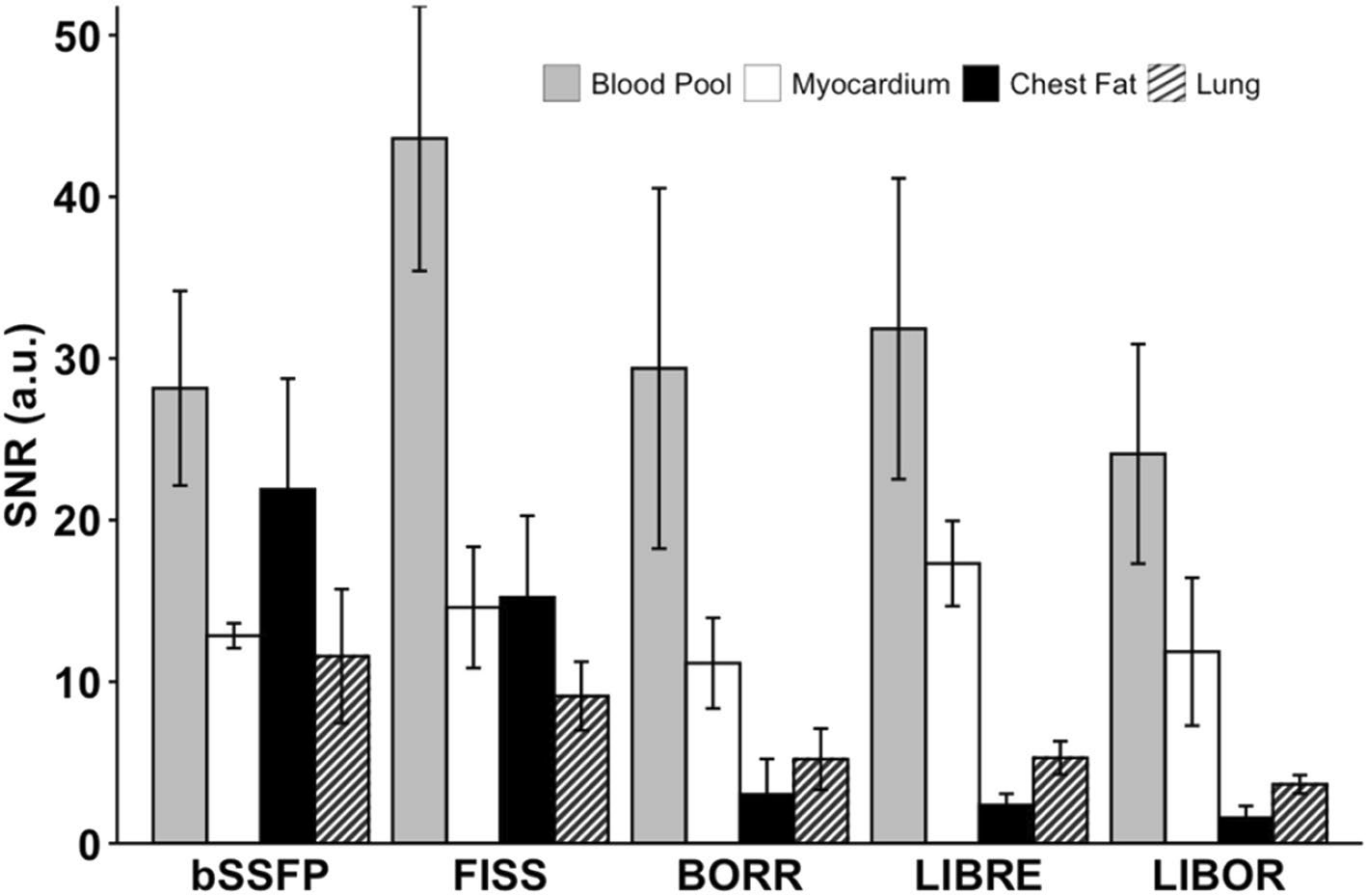
Signal-to-noise ratio (SNR) for volunteer experiments using different acquisitions. SNR was quantified for blood pool (gray), myocardium (white), chest fat (black), and lung (hatched) in images reconstructed using reconstruction without motion correction. Regions of interest (ROIs) were manually placed in the septal myocardium, left ventricular blood pool, chest fat, right lung, and background on axial slices for all tested sequences. Error bars represent the standard deviation across the cohort

Meanwhile, when using the reconstructed images without motion correction from the volunteers, BORR exhibited the highest SNR in the ventricular blood pool (17.0 ± 1.5), whereas LIBRE had the lowest value (13.5 ± 1.2). Comparing the SNR for chest fat, BORR had the highest (4.7 ± 2.2), and LIBRE had the lowest (3.6 ± 1.5) values (Fig. 9). The results showed no significant differences between acquisitions for the SNR of the blood pool (*p* = 0.565) and myocardium (*p* = 0.465). However, there was a significant difference in the SNR for chest fat compared to bSSFP (*p* = 0.0028).

**Fig. 10:**
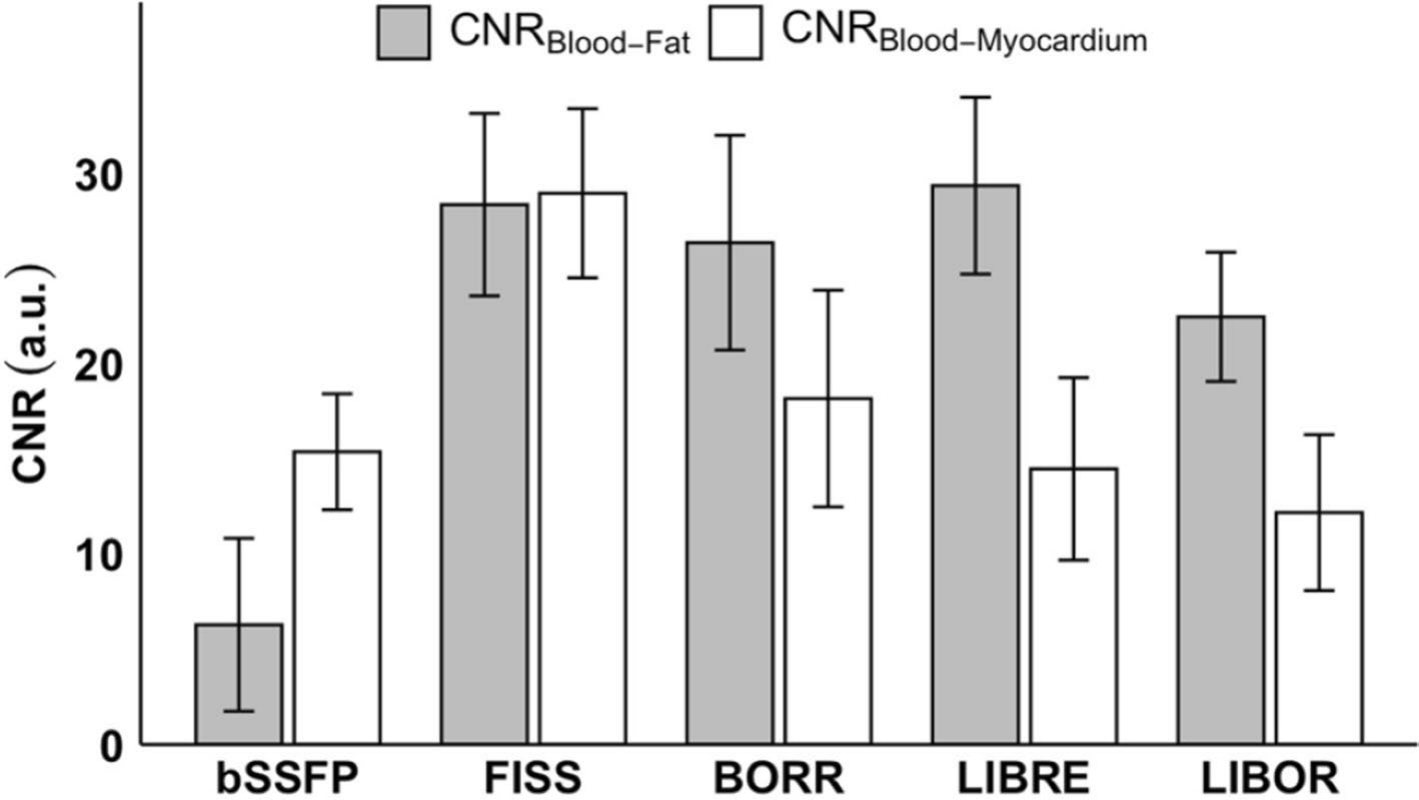
Contrast to Noise Ratios (CNR) for volunteer experiments using different sequences. The CNR_Blood-Fat_ (grey) was calculated by subtracting SNR_Blood Pool_ from SNR_Chest Fat_, and CNR_Blood-Myocardium_ (white) was calculated by subtracting SNR_Blood Pool_ from SNR_Myocardium_. All CNR values were obtained using reconstruction without motion correction. The sequences analyzed include conventional non-fat-suppressed bSSFP, FISS, BORR-bSSFP, LIBRE-bSSFP, and LIBOR-bSSFP pulses. Error bars represent the standard deviation across the cohort

When comparing the contrast between the blood pool and chest fat, LIBRE displayed the highest CNR_Blood-Fat_ (29.4 ± 9.3), in comparison to bSSFP (6.3 ± 9.1), FISS (28.4 ± 9.6), BORR (26.4 ± 9.3), and LIBOR (22.5 ± 6.8). FISS had the highest CNR_Blood-Myocardium_ (29.0 ± 8.9) compared to bSSFP (15.4 ± 6.1), BORR (18.2 ± 11.4), LIBRE (14.5 ± 9.6), and LIBOR (12.2 ± 8.2) (Fig. 10).

Overall, LIBOR had the lowest SAR. The recorded SAR increase in comparison with LIBOR was 6.8 in bSSFP, 6.1 with FISS, 3.3 with BORR, and 2.5 with the use of LIBRE RF pulses (Fig. 7).

## Discussion

This study compared different methods for fat signal suppression, such as FISS, and rapid off-resonant water-excitation pulses for free-running whole-heart MRI at 1.5 T in numerical simulations, phantom and volunteer experiments. The off-resonant water excitation pulses BORR and LIBOR were implemented and tested for the first time at 1.5 T, demonstrating promising results for whole-heart MRI using the free-running framework. All tested methods were comparable in terms of fat signal suppression but had different SAR depositions.

As expected based on recent findings at 3 T [20], the LIBOR pulse resulted in a significant reduction in RF energy deposition both in phantom and volunteer studies while maintaining comparable fat signal suppression. This makes using a LIBOR pulse particularly useful for acquisitions requiring a short TR and high SAR intensity, such as free-running sequences based on bSSFP. Its ability to provide robust fat suppression while maintaining low RF power deposition enhances image quality and could potentially improve diagnostic accuracy, particularly in cases where clear differentiation between fat and water signals is crucial, such as fat infiltration or lipomatous hypertrophy, where effective fat suppression can significantly enhance the visibility of subtle pathological features.

Although the BORR pulse had the highest SAR when compared with LIBOR and LIBRE, it had the broadest fat suppression bandwidth, which was also observed at 3 T [20, 25]. Therefore, the BORR pulse would benefit sequences that are less SAR intensive, for example, bSSFP sequences that are interrupted by ECG triggering or GRE sequences [16, 18].

Similar fat suppression was observed in numerical simulations and phantom experiments across the tested methods, but differences in fat signal around the heart and the chest could be observed in the volunteer experiments when comparing FISS with the water excitation methods. These are likely due to magnetic field inhomogeneities that are increased in the large imaging volumes in whole-heart MRI. The numerical simulations furthermore demonstrate that the excitation and suppression profiles are not homogeneous. Free-running multiecho GRE experiments could be performed to obtain a B_0_ map to quantify the fat signal within a voxel as function of off-resonance [12, 39]. Additional volunteer studies, with varying body composition, and inclusion of B_0_ maps would provide more insight in the actual fat suppression characteristics in different anatomical locations.

Optimal blood-myocardium contrast in bSSFP is typically achieved with RF excitation angles between 60°﻿ and 70°. Therefore, the nominal RF excitation angle of 50º caused a suboptimal CNR_Blood-Myocardium_ in volunteers. Due to SAR constraints encountered using BORR, the nominal RF excitation angles were capped at 50° to ensure a fair comparison among the different methods. When using LIBRE and LIBOR in future studies, the RF excitation angles should be increased independently to maximize the SNR, CNR, and image quality.

The FISS sequence resulted in the highest SAR, which may be reduced by prolonging the rectangular RF pulse duration from 0.3 ms to 0.5 ms. However, since specific TR values are essential for fat signal suppression, prolonging the TR due to an increase in RF pulse duration may negatively affect fat suppression [22]. Although the SAR was the highest, it is not prohibitive, and the FISS sequence provides a shorter scan time compared with the bSSFP sequence using water excitation pulses. FISS balances acquisition speed and resolution while maintaining manageable SAR levels [22]. FISS has been successfully used in various applications [23, 24], including as anatomical reference for flow MRI [40].

The spatial resolution used in this study was well-suited for cardiac cine imaging, which captures the heart in motion throughout its cardiac cycle. Due to the scan time constraints, incorporating a full stack of 2D cine acquisitions as a reference method was not possible. However, recent work demonstrated that cardiac cine imaging using the free-running framework [41], provided accurate calculations of ejection fraction, ventricular volumes, and cardiac mass. Whole-heart free-running cine imaging is therefore a feasible application of this work.

All tested methods behaved similarly in terms of fat-signal suppression. Streaking artifacts, which can result from inadequate fat suppression in non-Cartesian readouts, were not prominently observed in the volunteer scans. LIBOR emerged as a promising off-resonant water excitation pulse with the lowest SAR deposits, but with a longer scan duration compared with FISS. LIBOR demonstrated potential for functional cardiac imaging at high spatial resolutions similar to FISS and LIBRE [19, 22], offering a streamlined workflow compared to conventional CMR protocols within the free-running framework. The LIBOR pulse enhanced the contrast between the myocardium and surrounding tissues, ensuring clearer visualization of cardiac structures.

## Conclusion

Different fat signal suppression methods were evaluated for free-running whole-heart MRI at 1.5 T, marking the first report on both LIBOR and BORR pulses. LIBOR deposited the minimum RF power compared to LIBRE, BORR, and FISS, while maintaining adequate fat suppression with sufficient blood-muscle contrast. FISS demonstrated increased SAR but with reduced scan times.

## Data Availability

The datasets supporting the findings of this study are openly available in Zenodo at 10.5281/zenodo.13868461. The data have been deposited under an open license, ensuring accessibility for further research and enabling replication and validation of the study’s results.

## Abbreviations

2D: Two-dimensional
ADMM: Alternating direction method of multipliers
BORR: Binomial off-resonant rectangular
bSSFP: Balanced steady-state free precession
CMR: Cardiac magnetic resonance
CNR: Contrast-to-noise ratio
CS: Compressed sensing
DICOM: Digital imaging and communications in medicine
FISS: Fast interrupted steady-state
FOV: Field of view
GRE: Gradient recalled echo
LIBOR: Lipid insensitive binomial off-resonant
LIBRE: Lipid insensitive binomial off-resonant RF excitation
LLR: Local low-rank
RF: Radiofrequency
ROI: Region of interest
SAR: Specific absorption rate
SNR: Signal-to-noise ratio
TE: Echo time
TR: Repetition time
TV: Total variation
WE: Water excitation
